# The Carotenoid Esterification Gene *BrPYP* Controls Pale-Yellow Petal Color in Flowering Chinese Cabbage (*Brassica rapa* L. subsp. *parachinensis*)

**DOI:** 10.3389/fpls.2022.844140

**Published:** 2022-05-03

**Authors:** Peirong Li, Sirui Lv, Deshuang Zhang, Tongbing Su, Xiaoyun Xin, Weihong Wang, Xiuyun Zhao, Yangjun Yu, Yaowei Zhang, Shuancang Yu, Fenglan Zhang

**Affiliations:** ^1^Beijing Vegetable Research Center, Beijing Academy of Agriculture and Forestry Sciences, Beijing, China; ^2^Key Laboratory of Biology and Genetic Improvement of Horticultural Crops (North China), Ministry of Agriculture, Beijing, China; ^3^Beijing Key Laboratory of Vegetable Germplasm Improvement, Beijing, China; ^4^Key Laboratory of Biology and Genetic Improvement of Horticultural Crops (Northeast Region), Ministry of Agriculture, Harbin, China; ^5^College of Horticulture and Landscape Architecture, Northeast Agricultural University, Harbin, China

**Keywords:** carotenoid esterification, pale yellow petals, phytyl ester synthase, plastoglobules, flowering Chinese cabbage

## Abstract

Carotenoid esterification plays indispensable roles in preventing degradation and maintaining the stability of carotenoids. Although the carotenoid biosynthetic pathway has been well characterized, the molecular mechanisms underlying carotenoid esterification, especially in floral organs, remain poorly understood. In this study, we identified a natural mutant flowering Chinese cabbage (Caixin, *Brassica rapa* L. subsp. *chinensis var. parachinensis*) with visually distinguishable pale-yellow petals controlled by a single recessive gene. Transmission electron microscopy (TEM) demonstrated that the chromoplasts in the yellow petals were surrounded by more fully developed plastoglobules compared to the pale-yellow mutant. Carotenoid analyses further revealed that, compared to the pale-yellow petals, the yellow petals contained high levels of esterified carotenoids, including lutein caprate, violaxanthin dilaurate, violaxanthin-myristate-laurate, 5,6epoxy-luttein dilaurate, lutein dilaurate, and lutein laurate. Based on bulked segregation analysis and fine mapping, we subsequently identified the critical role of a phytyl ester synthase 2 protein (*PALE YELLOW PETAL*, *BrPYP*) in regulating carotenoid pigmentation in flowering Chinese cabbage petals. Compared to the yellow wild-type, a 1,148 bp deletion was identified in the promoter region of *BrPYP* in the pale-yellow mutant, resulting in down-regulated expression. Transgenic Arabidopsis plants harboring beta-glucuronidase (GUS) driven by yellow (*BrPYP*^Y^*::GUS*) and pale-yellow type (*BrPYP*^PY^*::GUS*) promoters were subsequently constructed, revealing stronger expression of *BrPYP*^Y^*::GUS* both in the leaves and petals. Furthermore, virus-induced gene silencing of *BrPYP* significantly altered petal color from yellow to pale yellow. These findings demonstrate the molecular mechanism of carotenoid esterification, suggesting a role of phytyl ester synthase in carotenoid biosynthesis of flowering Chinese cabbage.

## Introduction

Carotenoids play a number of vital roles in higher plant growth and development, harvesting light for photosynthesis (Hermanns et al., 2020) and protecting photosynthetic apparatus from photooxidation (Niyogi and Truong, 2013), as well as providing precursor metabolites for the biosynthesis of abscisic acids (ABA) and strigolactones (SLs) (Jia et al., 2018). The presence of carotenoids is also an evolutionary strategy that results in colorful petals and fruit, thereby acting to attract pollinators (Yuan et al., 2015).

Flower color, which is narrowly defined as petal color, is an essential trait of crops and vegetables with characteristics of phenotype variation, genetic stability and environmental adaptability (Rahman, 2001). It also plays an important role in hybrid seed purity by removing mixed non-target plant material and revealing the reliability of interspecific trait transfer (Zhang X. et al., 2018). Carotenoids range in color from yellow to red, resulting in a variety of eye-catching flower colors (Nisar et al., 2015). Carotenoid pigments are typically synthesized and stored in chromoplasts, a type of non-photosynthetic plastid (Sadali et al., 2019). Chromoplasts are characterized by the accumulation of plastoglobules (PGs), which contain a large quantity of xanthophyll esters (Mulisch and Krupinska, 2013). However, the role of chromoplasts in petal coloration is yet to be explored.

The concentration of carotenoid is co-regulated by its biosynthesis, degradation, and sequestration (Galpaz et al., 2006; Tanaka and Ohmiya, 2008; Yoshioka et al., 2012). Biosynthesis begins with isoprene precursors generated by the methylerythritol phosphate (MEP) pathway, with the subsequent reaction producing isopentenyl pyrophosphate (IPP) and its isomer dimethylallyl pyrophosphate (DMAPP) (Rodriguez-Concepcion and Boronat, 2015). Phytoene is the first carotenoid product, and is catalyzed from geranylgeranyl pyrophosphate (GGPP) by phytoene synthase (*PSY*) then converted into red lycopene *via* a series of desaturation and isomerization reactions (Cazzonelli and Pogson, 2010). Cyclization of lycopene by lycopene ε-cyclase (*LycE*) and/or lycopene β-cyclase (*LycB*) produces α-carotene and β-carotene, which are further hydroxylated to produce xanthophylls, such as lutein and zeaxanthin (Sun et al., 2018; Hermanns et al., 2020). Xanthophyll esterification effectively prevents carotenoid degradation and subsequently increases total levels within the chromoplasts (Ariizumi et al., 2014). However, little is known about the factors underlying xanthophyll esterification in chromoplasts and other higher plant plastids.

*Brassica rapa* crops belong to the Cruciferae family, which includes a number of economically important vegetables and oil crops, especially in East Asia. Flower color plays a significant role in seed purity, removing miscellaneous plants, and aiding the selection and breeding of *Brassica* varieties (Li et al., 2015). Recently, a number of genes controlling white and orange flower color were reported in *Brassica* crops. For example, the carotenoid isomerase gene *CRTISO* and plastid-lipid associated gene *PAP* were found to be related to orange and white flower color in Chinese cabbage, respectively (Li et al., 2015; Zhang et al., 2020). Furthermore, the carotenoid cleavage dioxygenase 4 gene (*CCD4*) was found to be responsible for the white flower color in several *B. oleracea* subspecies, including cabbage (Han et al., 2019; Zhang et al., 2019) and kale (Xu et al., 2019). Moreover, *BjuA008406* and *BjuB027334* were identified as candidate genes of white flower color in *B*. *juncea* (Zhang X. et al., 2018). Recently, the white flower gene *BrWF3*, which might associate with the production of xanthophyll esters, was identified based on the fine mapping and carotenoid analysis (Yang et al., 2021). However, the underlying molecular mechanism in *B. rapa* remains poorly understood.

In this study, we identified a natural mutant flowering Chinese cabbage plant (Caixin, *B. rapa* L. subsp. *Chinensis* var. *parachinensis*) with visually distinguishable pale-yellow petals. Inheritance pattern analysis followed by transmission electron microscopy (TEM) assay and carotenoid profiling were subsequently performed. Based on fine mapping and virus-induced gene silencing, we revealed that a phytyl ester synthase 2 protein [*PALE YELLOW PETAL* (*BrPYP*)] is responsible for the mutant pale-yellow petal color. Staining of transgenic Arabidopsis plants harboring beta-glucuronidase further revealed the function of the *BrPYP* promoter in regulating petal carotenoid pigmentation in flowering Chinese cabbage. Overall, our findings demonstrate the molecular mechanism underlying *BrPYP*-mediated regulation of petal carotenoid accumulation, and suggest a role of phytyl ester synthase in carotenoid esterification in *B. rapa*.

## Materials and Methods

### Plant Materials

Inbred lines of the pale-yellow parent (20M-293, P_1_) and yellow parent (20M-294, P_2_) were used in all experiments. P_1_ was crossed with P_2_ to produce the hybrid F_1_ then the F_1_ was self-pollinated to obtain the F_2_ population. The F_1_ plants were also backcrossed with 20M-293 to produce a BC_1_ population. All materials were grown in a greenhouse at the Beijing Vegetable Research Center.

### Identification of Candidate Genes Using Bulk Segregation Analysis and Kompetitive Allele-Specific PCR Assay

Candidate genes were identified according to the BSA method (Takagi et al., 2013). Fifty yellow and 50 pale-yellow individuals from the F_2_ population were selected for each pool. Equal amounts of DNA from the selected individuals were then combined and DNA libraries were prepared using a TruSeq Library Construction Kit before sequencing on the HiSeq™ PE150 platform (Illumina, San Diego, United States).

The raw reads were filtered by removing reads with adapters, reads with an N value greater than 10%, and low-quality reads with a quality value *Q* ≤ 10. The clean reads were then aligned to the *Brassica rapa* reference genome V3.0 (Zhang L. et al., 2018) using Burrows-Wheeler Aligner (BWA) (Li and Durbin, 2010). Detection of SNPs and InDels was carried out using a GATK software toolkit (McKenna et al., 2010) with default parameters. The average ΔSNP-index across 10 chromosomes in the *B. rapa* genome was measured individually using the sliding-window approach, with a 2 Mb window size and a 10 kb sliding-window step.

High−quality SNP candidates between two parents were selected for KASP marker development. For each SNP, two allele-specific forward primers and one common reverse primer were designed by LGC Genomics (Supplementary Table 1). The amplifications and analysis methods were performed based on the previously published guidelines (Li et al., 2019).

### Gene Cloning and Sequence Analysis

Total genomic DNA was extracted from fresh petals using the cetyl trimethylammonium bromide (CTAB) method (Chen and Ronald, 1999). The reference sequences of *BraA02g037160.3C* and *BraA02g037170.3C* were obtained from the *B. rapa* database, and used to design gene-specific and promoter primers (Supplementary Table 2). The cloning and sequencing of candidate genes were performed as described previously (Li et al., 2021).

The sequences of *BraA02g037170.3C* were then aligned using ClustalW^1^ and displayed using ESPript^2^ The cis- and trans-acting elements of the *BraA02g037170.3C* promoter region were also predicted using the PlantCARE^3^ online analysis software program.

### RNA Extraction and Expression Analysis

Total RNA was extracted from petals of the two parent lines using the RNAprep Pure Plant Plus Kit (TIANGEN, Beijing, China). First-strand cDNA, which was used for real-time PCR (RT-PCR) and quantitative real-time PCR (qRT-PCR), was synthesized using the PrimeScript RT Reagent Kit with gDNA Eraser (TaKaRa, Japan). Specific primers of *BraA02g037160.3C*, *BraA02g037170.3C*, and two phytoene desaturase (*PDS*) genes for RT-PCR and qRT-PCR were designed using the Primer Premier 6.0 software. PCR amplification and conditions were as described previously (Li et al., 2020). Gene expression was normalized using the housekeeping gene *GAPDH*. Reactions were performed with three biological replicates and three technical replicates, and transcript levels in different colored flowers were analyzed using the 2^–Δ^ ^Δ^ *^Ct^* method (Livak and Schmittgen, 2001). Primer sequences are listed in Supplementary Table 3.

### Carotenoid Detection

We detected carotenoid contents by Metware^4^ based on the AB SCIEX QTRAP 6500 liquid chromatography with tandem mass spectrometry (LCMS/MS) platform. Three biological replicates of each parent were assessed. In brief, samples from pale-yellow and yellow petals were freeze-dried then stored at -80°C until use. The dried samples were then homogenized in a grinder before extracting 50 mg of dried powder with a solution of n-hexane: acetone: ethanol. An internal standard was then added and the extract was centrifuged for 20 min at room temperature. The supernatants were then collected and the above steps were repeated to re-extract the residue. The extract was then evaporated to dryness under nitrogen flow and reconstituted in methanol: MTBE solution then filtered through a 0.22 μm filter for LC-MS analysis.

The analytical conditions were as follows: column, YMC C30 (3 μm, 100 mm × 2.0 mm); solvent system, methanol: acetonitrile (1:3, V/V) with 0.01% BHT and 0.1% formic acid (A), methyl tert-butyl ether with 0.01% BHT (B); gradient program, started at 0% B (0–3 min), increased to 70% B (3–5 min), then increased to 95% B (5–9 min), finally ramped back to 0% B (10–11 min); flow rate, 0.8 mL/min; temperature, 28°C; injection volume: 2μL.

AB 6500 Q TRAP LC/MS/MS System, equipped with an APCI Heated Nebulizer interface, operating in a positive ion mode, and controlled by Analyst 1.6.3 software (AB Sciex). The APCI source operation parameters were as follow: ion source, APCI +; source temperature 350°C; curtain gas (CUR) was set at 25.0 psi. DP and CE for individual MRM (Multiple Reaction Monitoring) transition was done with further DP and CE optimization. A specific set of MRM transitions were monitored for each period according to the carotenoids eluted within this period.

### Virus-Induced Gene Silencing Assay

To determine the efficiency of the VIGS technique in *B. rapa*, the *PDS* gene, which can be easily used to score the photobleached phenotype, was used as a visual marker. The hairpin sequence and plasmid construction of *BrPDS* were performed based on the previously published protocol (Yu et al., 2018). An appropriate fragment of 40 nt of *BrPYP* was then selected and a 80-nt palindromic DNA fragment of with *Sna*BI restriction sites and homologous arms on both sides was designed (TTAAACTACTTAGCTCCGTCTTCGGGCTCTCTCCTGTCT CGAGACAGGAGAGAGCCCGAAGACGGAGCTAAGTAGTT TAA). The palindromic DNA fragment was produced by Nanjing Genscript Technology Company (Nanjing, China) and linked to pTYs using the ClonExpress Entry One Step Cloning Kit (Vazyme) (pTYsBrPDS and pTYsBrPYP). The plasmid was then transformed into competent Super Stbl3 (Beijing, Huayueyang) on an Ampicillin (50 μg/ml) resistant plate then cultured overnight before selecting single clones. The clones were then placed in 1 L LB liquid medium and shaken at 225 rmp at a temperature of 30°C for 16 h. The plasmid sample was extracted using a Plasmid Giga Kit (OMEGA, United States) then the working concentration was adjusted to 300 μg/μl. A 5 μl leaf sample was then selected, rubbed with quartz sand, left to stand for 1 min then washed with water. The second vaccination was carried out 4–5 days later. Plants used for the VIGS assay were grown in a greenhouse with a temperature regimen of 25°C day/18°C night. Bacterial solution was then infiltrated into the leaves to obtain the silencing effect.

### Glucuronidase Staining and Activity

A sequence approximately 2 kb upstream of the gene start codon was used as a template for the cloned gene promoter sequence. Specific PCR primers were designed, and the target fragment was amplified then connected to the PBI121 (HindIII/BamhI) vector *via* homologous recombination. After transforming DH5α, a single clone was selected then sequenced before extracting the plasmid and transforming it into Agrobacterium GV3101. After plaque detection, *A. thaliana* was transformed using the floral dipping method (Clough and Bent, 1998). Seeds from the transformed Arabidopsis plants were then screened on 1/2 MS medium supplemented with 1.5% (w/v) sucrose and 0.8% (w/v) agar containing 25 mg/L of kanamycin (Yue et al., 2019). GUS staining of positive plants was performed using GUS staining solution at 37°C for 24 h with a GUSBlue KIT (Beijing Huayueyang, China). They were then decolorized with 75% ethanol and observed under a stereoscope (Olympus, Japan).

## Results

### Phenotypic Characterization of Pale-Yellow and Yellow Petals in Flowering Chinese Cabbage

**FIGURE 1:**
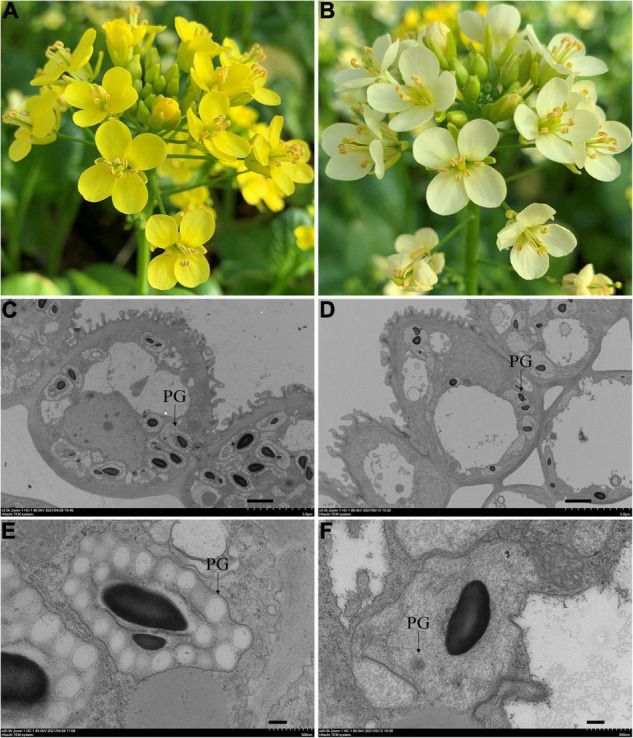
Phenotypes of the yellow and pale-yellow flowering Chinese cabbage petals. Yellow **(A)** and pale-yellow flower petals **(B)**. Plastid morphology in the yellow **(C,E)** and pale-yellow petals **(D,F)** was compared by TEM analysis. Enlarged images are also shown **(E,F)**. PG: plastoglobules. Scale bars: **(C,D)** 2 μm, **(E,F)** 200 nm.

Natural mutant flowering Chinese cabbage plants with visually distinguishable pale-yellow flowers were found in the field (Figures 1A,B). To clarify the inheritance pattern, we constructed two populations using a pale-yellow parent (P_1_) and yellow parent (P_2_) (Figures 1A,B). As expected, the flowers of F_1_ plants derived from a reciprocal cross between the two parents were all yellow. Of 442 F_2_ individuals, 343 had yellow flowers and 99 had pale yellow flowers. A χ^2^-test subsequently confirmed the F_2_ segregation ratio to be 3:1 (χ^2^_0.05_ = 3.84; *P* > 0.05; Table 1). Furthermore, flower color was also examined in a BC_1_ population derived from F_1_ individuals backcrossed with the pale yellow-flowered parent, revealing an expected Mendelian inheritance ratio of 1 yellow: 1 pale yellow (χ^2^_0.05_ = 3.84; *P* > 0.05; Table 1). These results indicated that the pale-yellow flower trait is controlled by a single recessive *BrPYP* gene (Table 1).

**TABLE 1:** The segregation of flower colors in the F_2_ and BC_1_ population.

Generation	Total plants	Yellow flower plants	White flower plants	Goodness of fit X^2^
F_2_	442	343	99	1.46
BC_1_	1,002	521	481	1.51

*X^2^ > X^2^_0_._05_ = 3.84 is considered significant.*

Since carotenoids are biosynthesized and stored in chromoplasts, TEM was used to determine whether the pale-yellow color was due to changes in the morphology of the chromoplasts. Accordingly, chromoplasts in the yellow petals were well developed and surrounded by numerous fully developed PGs (Figures 1C,E). However, in contrast, chromoplasts in the pale-yellow petals only contains far fewer well-developed PGs (Figures 1D,F).

### Carotenoid Analysis of Pale-Yellow and Yellow Flowers Using LC-MS/MS

To examine whether the petal coloration was due to a reduction in carotenoid accumulation, carotenoid components were analyzed in three pale-yellow and three yellow flowers. Ultra-performance liquid chromatography-tandem mass spectrometry (LC-MS/MS) analysis was used to identify non-esterified and esterified carotenoids (Table 2). As a result, a total of 34 carotenoid compounds were differently accumulated between yellow and pale-yellow petals (Table 2), 28 and 25 of which were found in the pale-yellow and yellow petals, respectively. Of these, 22 compounds were less abundant in the pale-yellow petals, including nine compounds that were not detected (Table 2).

**TABLE 2:** Difference of carotenoids accumulation and their derivative esters present in yellow and pale-yellow petals.

Compounds	Class	Yellow (μ g/g)	Pale-yellow (μ g/g)	Log2FC	Type
Lutein caprate	Carotenoid esters	266 ± 5	1.17 ± 0.09	-7.83	Down
(E/Z)-Phytoene	Carotenes	89.7 ± 7.5	32.27 ± 5.81	-1.47	Down
Violaxanthin dilaurate	Carotenoid esters	46.97 ± 5.99	0.41 ± 0.1	-6.84	Down
Violaxanthin-myristate-laurate	Carotenoid esters	17.03 ± 3.35	NA	NA	Down
5,6epoxy-luttein dilaurate	Carotenoid esters	15.87 ± 2.76	0.16 ± 0.06	-6.63	Down
Lutein dilaurate	Carotenoid esters	8.89 ± 1.63	1.09 ± 0.05	-3.03	Down
Lutein laurate	Carotenoid esters	6.61 ± 0.31	NA	NA	Down
β-Cryptoxanthin	Xanthophylls	5.96 ± 0.39	2.83 ± 0.11	-1.07	Down
Rubixanthin caprate	Carotenoid esters	3.35 ± 0.64	NA	NA	Down
α-Carotene	Carotenes	2.25 ± 0.27	NA	NA	Down
α-Cryptoxanthin	Xanthophylls	2.24 ± 0.15	0.84 ± 0.15	-1.42	Down
Neochrome palmitate	Carotenoid esters	1.9 ± 0.28	0.84 ± 0.33	-1.18	Down
β-Cryptoxanthin laurate	Carotenoid esters	1.59 ± 0.28	0.11 ± 0.02	-3.85	Down
5,6-epoxy-lutein-caprate-palmitate	Carotenoid esters	1.48 ± 0.44	NA	NA	Down
Violaxanthin palmitoleate	Carotenoid esters	1.09 ± 0.22	NA	NA	Down
γ-Carotene	Carotenes	0.7 ± 0.17	NA	NA	Down
Rubixanthin palmitate	Carotenoid esters	0.54 ± 0.08	0.19 ± 0.01	-1.51	Down
β-Cryptoxanthin palmitate	Carotenoid esters	0.52 ± 0.1	0.23 ± 0.02	-1.18	Down
Zeaxanthin dilaurate	Carotenoid esters	0.19 ± 0.04	0.01 ± 0	-4.25	Down
β-Cryptoxanthin oleate	Carotenoid esters	0.11 ± 0	NA	NA	Down
Echinenone	Xanthophylls	0.04 ± 0	0.01 ± 0	-2.00	Down
Canthaxanthin	Xanthophylls	0.01 ± 0	NA	NA	Down
Apocarotenal	Xanthophylls	NA	0.03 ± 0	NA	Up
ε-Carotene	Carotenes	NA	0.15 ± 0.02	NA	Up
Antheraxanthin dipalmitate	Carotenoid esters	NA	0.33 ± 0.1	NA	Up
Violaxanthin laurate	Carotenoid esters	NA	0.36 ± 0.03	NA	Up
Violaxanthin dipalmitate	Carotenoid esters	NA	0.47 ± 0.09	NA	Up
Zeaxanthin dipalmitate	Carotenoid esters	NA	0.58 ± 0.15	NA	Up
Lutein dimyristate	Carotenoid esters	12.67 ± 0.9	24.25 ± 1.35	0.94	Up
Violaxanthin myristate	Carotenoid esters	1.56 ± 0.52	4.73 ± 0.96	1.60	Up
Violaxanthin palmitate	Carotenoid esters	1.02 ± 0.09	3.59 ± 0.8	1.82	Up
Violaxanthin-myristate-palmitate	Carotenoid esters	0.46 ± 0.03	7.85 ± 1.42	4.09	Up
Violaxanthin dimyristate	Carotenoid esters	0.4 ± 0.13	16.03 ± 2.84	5.32	Up
β-Citraurin	Xanthophylls	0.03 ± 0	0.08 ± 0.01	1.42	Up

*NA means that the substance is not detected in this assay.*

A comparison of carotenoid profiles between the pale-yellow and yellow petals demonstrated abundant esterified carotenoids in the yellow petals. Moreover, carotenoid esters, such as lutein caprate, violaxanthin dilaurate, violaxanthin-myristate-laurate, 5,6epoxy-luttein dilaurate, lutein dilaurate, and lutein laurate, were highly abundant or found only in the yellow petals (Table 2). Taken together, these results suggest that yellow petals accumulate more carotenoid esters (namely, lutein caprate, and violaxanthin dilaurate) than pale-yellow petals, resulting in greater carotenoid accumulation and subsequent yellow pigmentation.

### Identification of *BrPYP* Using Bulk Segregation Analysis and Fine Mapping

**FIGURE 2:**
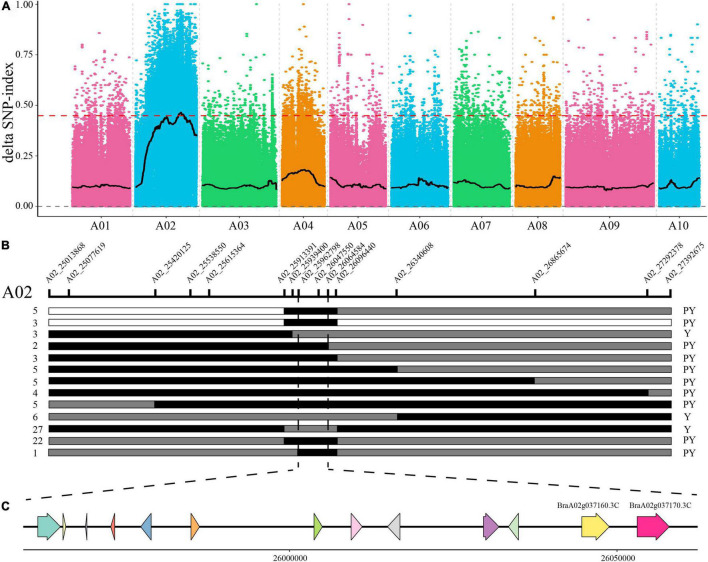
Fine mapping of the pale-yellow petal gene *BrPYP.* **(A)** SNP index plot of 10 chromosomes produced using BSA analysis. The x-axis represents the position of the 10 chromosomes, while the y-axis represents the SNP index. The dashed line represents the index threshold (0.99). **(B)** F_2_ and BC_1_ populations were applied to the *BrPYP* fine map, narrowing down the area to 101.79 kb. The SNP markers used for mapping are labeled in the schematic diagram of the chromosome. The number of recombinants within the same genotype are written on the left, with phenotypes on the right. “Y” and “PY” refer to yellow and pale-yellow phenotypes, respectively. White and gray segments indicate the same genotype as the yellow parent and F_1_, respectively, while the black segment represents the same genotype as the pale-yellow parent. **(C)** The 13 genes in the candidate region.

To identify candidates of the *BrPYP* gene, 50 pale yellow and 50 yellow individuals were selected from the F_2_ population and used to construct extreme pools for bulk segregation analysis (BSA). Genome resequencing of the pale-yellow parent (P_1_), yellow parent (P_2_) and two DNA pools (pale-yellow and yellow bulk) resulted in 202,531,626, 208,423,334, 209,006,868, and 207,592,868 high-quality clean reads, respectively (Supplementary Table 4). Meanwhile, 98.12, 98.35, 98.52, and 98.57% of the clean reads (Supplementary Table 5) were, respectively, aligned to the reference genome. Using a single-nucleotide polymorphism (SNP) index of 0.99 as the threshold, we were able to preliminarily map *BrPYP* to a candidate region of 2.76Mb (24,960,000–27,720,000) on chromosome A02 (Figure 2A).

To fine map *BrPYP*, we used both BC_1_ and F_2_ individuals to conduct Kompetitive Allele-Specific PCR (KASP) genotyping analysis. SNPs were acquired based on the resequencing results with 50 SNP markers in the candidate region initially used to identify recombinant individuals (Figure 2B). Subsequently, 15 SNP markers exhibiting polymorphisms were detected between the pale-yellow bulk and yellow bulk (Supplementary Table 1). Finally, *BrPYP* was localized to a 101.79 kb region between SNP markers A02_25962798 and A02_26064584 (Figure 2B). Specifically, the SNP marker within the *BraA02g037170.3C*, A02_26057775 gene co-segregated with the pale-yellow flower trait.

### Candidate Gene Cloning, Sequencing and Expression Analysis

To identify *BrPYP* in flowering Chinese cabbage, comparative gene annotation was carried out in *Arabidopsis thaliana*. Overall, 13 genes were identified in the candidate region, of which nine possessed functional annotations suggestive of Arabidopsis homologs (Table 3). However, among these, no carotenoid biosynthesis genes were identified. We therefore screened each gene according to previously published data, revealing two homologs of *Phytyl ester synthase 2* (*PES2*) as candidates. *PES2* encodes a carotenoid modifying protein, which plays a vital role in the production of xanthophyll esters in anthers and petals (Ariizumi et al., 2014).

**TABLE 3:** Thirteen annotated genes in the candidate region of *BrPYP*.

Gene	Chr	Start	End	Arabidopsis ID	Annotation
*BraA02g037050.3C*	A02	25,961,578	25,965,034	*AT3G26730*	E3 ubiquitin-protein ligase RMA1H1-like
*BraA02g037060.3C*	A02	25,965,421	25,965,848	-	Light-regulated protein 1, LIR1
*BraA02g037070.3C*	A02	25,968,852	25,969,076	-	tRNA (guanine (10)-N2)-methyltransferase homolog
*BraA02g037080.3C*	A02	25,972,710	25,973,315	-	Glycine-rich RNA-binding protein GRP1A-like isoform X1
*BraA02g037090.3C*	A02	25,977,283	25,978,878	*AT3G26744*	Transcription factor ICE1, SCRM
*BraA02g037100.3C*	A02	25,984,959	25,986,267	*AT3G26760*	Short-chain dehydrogenase reductase 2a, SDR2a
*BraA02g037110.3C*	A02	26,003,742	26,005,011	*AT3G26770*	Short-chain dehydrogenase reductase 2a, SDR2a
*BraA02g037120.3C*	A02	26,009,406	26,011,108	*AT3G26780*	Aquaporin TIP1-2
*BraA02g037130.3C*	A02	26,014,958	26,016,857	*AT3G26790*	B3 domain-containing transcription factor FUS3, FUS3
*BraA02g037140.3C*	A02	26,029,608	26,031,989	*AT3G26810*	AUXIN SIGNALING F-BOX 2, AFB2
*BraA02g037150.3C*	A02	26,033,422	26,034,954	-	NIPA-like protein 2
*BraA02g037160.3C*	A02	26,044,622	26,048,832	*AT3G26840*	Acyltransferase-like protein
*BraA02g037170.3C*	A02	26,053,073	26,057,985	*AT3G26840*	Acyltransferase-like protein

**FIGURE 3:**
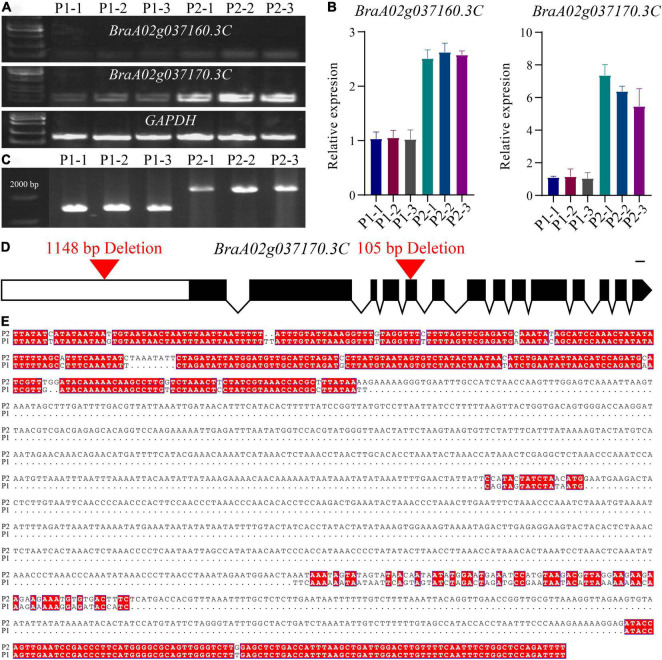
Molecular cloning and confirmation of the *BrPYP* gene. **(A)** RT-PCR showing transcript abundance of *BraA02g037160.3C* and *BraA02g037170.3C* in the pale-yellow (P_1_) and yellow petals (P_2_). *GAPDH* was used as a control. **(B)** qRT-PCR showing transcript levels of *BraA02g037160.3C* and *BraA02g037170.3C* in the P_1_ and P_2_ petals. **(C)** Promoter amplification showing a large deletion in *BraA02g037170.3C* of P_1_. **(D)** Schematic representation of the gene structure of *BrPYP* showing the deletion in the promoter and the fifth exon of the pale-yellow parent. Introns are represented by a line, and exons are represented by black boxes. **(E)** The promoter was then sequenced and aligned (from 978 bp upstream to 2,500 bp) between P_1_ and P_2_.

Quantitative real-time PCR (qRT-PCR) was subsequently performed to determine the expression level of *BraA02g037160.3C* and *BraA02g037170.3C* in the petals of the pale-yellow and yellow parent. Expression of both genes was lower in the pale-yellow mutant than the yellow parent (Figures 3A,B). However, in comparison, expression of *BraA02g037170*.*3C* was very high in the yellow parent and six-fold higher than in the pale-yellow parent. Meanwhile, expression of *BraA02g037160*.*3C* was only two-fold higher in the yellow parent than the pale-yellow parent, and was very low in both (Figure 3). To further characterize the difference between the two parents, the promoter of *BraA02g037170*.*3C* was subsequently amplified and sequenced. Sequence alignment revealed a 1,148 bp deletion starting 1,078 bp upstream of the promoter in the pale-yellow parent, with several other SNPs and InDels (Figures 3C,D). Analysis of Cis-acting regulatory elements further revealed that the deleted sequence contained multiple light-responsive motifs, including the TCT-motif, chs-CMA1a, MRE, GT1-motif, G-box, and ACE (Supplementary Table 6).

Analysis of cDNA sequences of *BraA02g037170*.*3C* from both parents indicated a 105-bp deletion in the fifth exon of the pale-yellow parent, resulting in a 35-amino acid deletion (from 219 to 253) (Supplementary Figure 1a). Furthermore, protein analysis demonstrated two domains based on the NCBI Conserved Domain Database (Supplementary Figure 1b): alpha/beta hydrolases and lysophospholipid acyltransferases (LPLATs), both of which are involved in glycerophospholipid biosynthesis. Alpha/beta hydrolases are a functionally diverse superfamily consisting of proteases, lipases, peroxidases, epoxide hydrolases, and esterases. Interestingly, the 35-amino acid deletion in pale-yellow allele was in alpha/beta hydrolases domain (Supplementary Figure 1), which may also affect the function of BrPYP protein.

### Silencing of *BrPYP* Causes a Pale-Yellow Petal Phenotype

**FIGURE 4:**
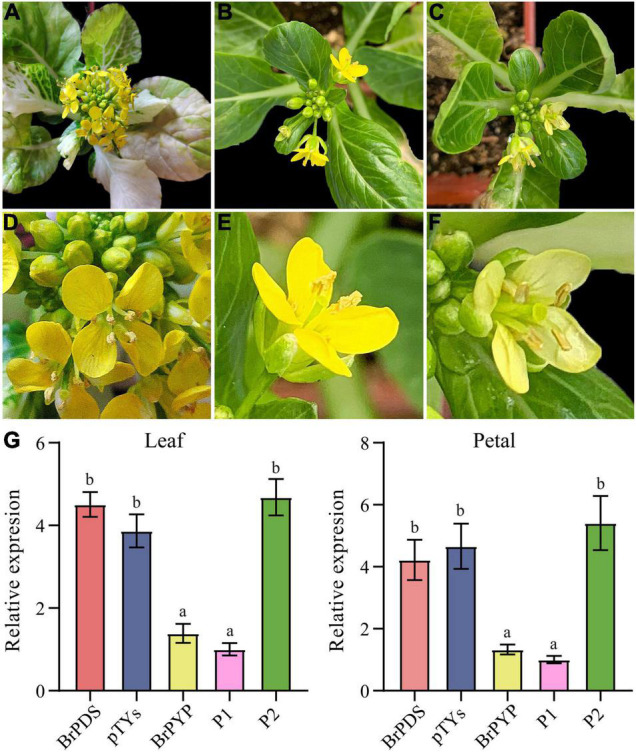
Transient silencing of *BrPYP* resulted in pale-yellow petals. **(A)** The evirus-induced gene silencing (VIGS)-mediated *BrPDS* gene silencing phenotype of flowering Chinese cabbage leaves. **(B)** Flowering Chinese cabbage plants were agro-drenched with empty pTYs vectors. **(C)** The VIGS-mediated *BrPYP* gene silencing phenotype of flowering Chinese cabbage petals. **(D–F)** Enlarged images of **(A–C)**, respectively. **(G)** qRT-PCR analysis of VIGS-mediated *BrPYP* in flowering Chinese cabbage leaves and petals. The letters above the bars indicate significant differences at *P* < 0.05 (Student’s t-test).

Virus-induced gene silencing (VIGS) is a transient gene knockdown system that has successfully been used to characterize genes with different functions in *B. rapa*. We therefore used the VIGS approach to determine whether *BrPYP* can induce pale-yellow petals. Loss of chlorophyll pigmentation, caused by silencing of the *PDS* gene, was used as a visual marker to demonstrate the usefulness of this technique in efficient silencing of *B. rapa* genes (Figure 4A). Notably, both pTYsBrPDS and pTYs showed the same yellow petals as the yellow petal parent (Figures 4A,B). *BrPYP* gene silencing was subsequently monitored during flowering, revealing that successful silencing resulted in the pale-yellow phenotype (Figure 4C). The enlarged images of these petals were shown in Figures 4D–F.

qRT-PCR was also performed to further confirm the function of *PYP* at the transcriptional expression level mediated by VIGS (Figure 4G). The relative expression demonstrated that the *BrPDS* genes were successfully reduced in the leaves of plants infected with pTYsBrPDS (Supplementary Figure 2). Accordingly, transcript levels of *BrPYP* were substantially reduced both in the leaves and petals of pTYsBrPYP compared with pTYsBrPDS, pTYs and the yellow parent control (Figure 4G). Meanwhile, transcript levels of the *BrPYP* gene in *BrPYP*-silenced plants, which were similar to the pale-yellow parent, showed an approximate 70% reduction compared with the PDS-inoculated plants. This further confirms that *BraA02g037170*.*3C* represents the *BrPYP* gene responsible for the pale-yellow petal trait in flowering Chinese cabbage.

### Expression Patterns of *BrPYP*

**FIGURE 5:**
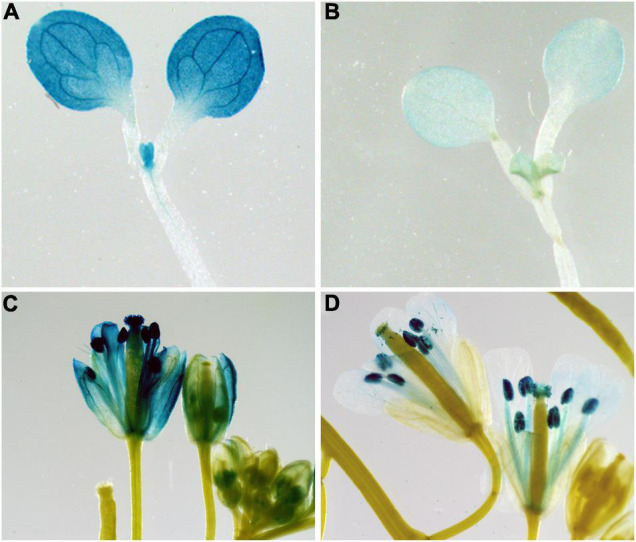
Expression patterns of *BrPYP* in *Arabidopsis*. *BrPYP* promoter-GUS expression of the yellow **(A)** and pale-yellow **(B)** type in *Arabidopsis* leaves. *BrPYP* promoter-GUS expression of yellow **(C)** and pale-yellow **(D)** type in *Arabidopsis* flowers.

To determine whether the promoter of *BrPYP* functions in carotenoid esterification, we constructed transgenic Arabidopsis plants harboring beta-glucuronidase (GUS) driven by the synthetic promoter *BrPYP* (Figure 5). We first analyzed the expression pattern of *BrPYP:GUS* in the Arabidopsis seedlings, revealing strong GUS activity in the leaves under the yellow petal promoter (*BrPYP*^Y^*::GUS*) compared with the pale-yellow petal promoter (*BrPYP*^PY^*::GUS*) (Figures 5A,B). As expected, GUS expression was also stronger in the petals of Arabidopsis with *BrPYP*^Y^*::GUS* (Figures 5C,D), indicating that variation in the promoter of pale-yellow petals results in a decrease in *BrPYP* expression.

## Discussion

### Insufficient Plastoglobules in the Pale-Yellow Petals of Flowering Chinese Cabbage Result in the Poor Accumulation of Carotenoids

This study demonstrated that the chromoplasts in the pale-yellow petals of flowering Chinese cabbage only contains far fewer well-developed PGs compared to the yellow petals (Figure 1). Chromoplasts are a type of plastid that participate in carotenoid pigment biosynthesis and storage through multiple catalytic steps (Sun et al., 2018; Hermanns et al., 2020). An increase in the number and size of mature chromoplasts leads to an accumulation of carotenoids, resulting in colorful petals that then act to attract pollinators (Li and Yuan, 2013). The PGs in chromoplasts are much bigger than those in chloroplasts and contain more xanthophyll esters, which are formed during the degradation of carotenoids (Mulisch and Krupinska, 2013). However, irregularly shaped PGs, and disorganized and electron-lucent membrane structures lead to a decrease in carotenoid, as found in carnations, tomato, and *B. juncea* (Ariizumi et al., 2014; Zhang X. et al., 2018; Iijima et al., 2020). In this study, far fewer well-developed PGs were observed in the pale-yellow petals of flowering Chinese cabbage, suggesting that insufficient PGs reduces accumulation of carotenoid esters.

### A Decrease in Carotenoid Esters Directly Affects Petal Color in Flowering Chinese Cabbage

(E/Z)-Phytoene and carotenoid esters, such as lutein caprate, violaxanthin dilaurate, violaxanthin-myristate-laurate, 5,6epoxy-luttein dilaurate, lutein dilaurate and lutein laurate, were highly accumulated or only found in the yellow flowering Chinese cabbage petals (Table 2). Carotenoids can be acylated by different fatty acids, generating a large number of possible carotenoid ester compounds (Schweiggert et al., 2016; Mariutti and Mercadante, 2018). Lutein esters, such as lutein caprate, 5,6epoxy-luttein dilaurate, lutein dilaurate and lutein laurate, are also the well-known predominant carotenoids in petals of marigold (Rodrigues et al., 2019) and tomato (Watanabe et al., 2021). Carotenoid esters are also generated by acylation of fatty acids (Fas), and esterification does not change the carotenoid chromophore; thus, all carotenoid esters present the same UV-VIS spectrum and color of free-carotenoid (Mercadante et al., 2017; Rodrigues et al., 2019). Interestingly, the pale-yellow flowering Chinese cabbage petals observed here had slightly more lutein dimyristate, violaxanthin myristate, violaxanthin-myristate-palmitate, and violaxanthin dimyristate than those of the yellow petals (Table 2). However, the absolute content of carotenoids in the pale-yellow petals was not very high, possibly resulting in the pale-yellow rather than pure white coloring. Based on the carotenoid profile detailed above, these findings suggest that the presence of lutein caprate and violaxanthin dilaurate are mainly responsible for the obvious color differences between yellow and pale-yellow flowering Chinese cabbage petals.

### *BrPYP* Is the Key Factor Causing Pale-Yellow Petals in Flowering Chinese Cabbage

In many plants, yellow petals generally contain high concentrations of carotenoids, consisting largely of esterified lutein (Meng et al., 2019). A decease in carotenoids accumulation and subsequent alteration in petal color from yellow to white or pale yellow can result from carotenoid biosynthesis, degradation or sequestration (Ariizumi et al., 2014; Meng et al., 2019). In *Medicago truncatula*, disruption of the anthocyanin-related R2R3-MYB protein WP1, which functions as a transcriptional activator directly regulating expression of the carotenoid biosynthetic genes *MtLYCe* and *MtLYCb*, was found to cause a reduction in lutein accumulation (Meng et al., 2019). Meanwhile, the tetratricopeptide repeat protein RCP2 was found to regulate carotenoid biosynthesis and chromoplast compartment size in Monkeyflowers, suggesting that loss-of-function mutations in *RCP2* damage the entire carotenoid biosynthetic pathway (Stanley et al., 2020). Plastid-localized CCDs are known to play significant roles in carotenoid degradation in many plants (Hai et al., 2012; Gonzalez-Jorge et al., 2013; Xu et al., 2019), which is negatively correlated with yellow pigment intensity, and is also part of the proteome of PGs. In addition to biosynthesis and degradation, carotenoid levels are also modulated by sequestration. Although the mechanisms involved in carotenoid sequestration remain elusive, several lines of evidence suggest the involvement of the phytyl ester synthase (PES) gene (Ariizumi et al., 2014; Yang et al., 2021). Disruption of *PES* caused a loss of xanthophyll esters, resulting in a decrease in total carotenoid levels and disruption of chromoplast development in the petals of plants (Ariizumi et al., 2014; Yang et al., 2021).

Our study strongly suggests that the *BrPYP* gene encodes an enzyme protein that controls the pale-yellow petal color in flowering Chinese cabbage (Figure 2). *BrPYP* is a homolog of Arabidopsis phytyl ester synthases 2 (*PES2*, *At3g26840*), a chloroplastic acyltransferase with phytyl ester synthesis and diacylglycerol acyltransferase activities (Lippold et al., 2012). Furthermore, *BrPYP* mapped to the same gene with *BrWF3*, which is a candidate gene responsible for white pigmentation in Chinese cabbage (*Brassica rapa* L. ssp. *pekinensis*) (Yang et al., 2021). However, different variations were identified between *BrPYP* and *BrWF3*. In the coding sequence of *BrWF3*, a SNP deletion was speculated to be key variant, causing a frameshift mutation and a premature stop codon (Yang et al., 2021). Meanwhile, we identified a functional 1,148 bp deletion in the promoter region of *BrPYP* that reduces promoter activity and expression level, and then verified by VIGS. As Caixin and Chinese cabbage are different subspecies of *B. rapa*, this could be different alleles between these two subspecies.

Fatty acid phytyl esters are a type of neutral lipid, the main compound of the PG core (Besagni and Kessler, 2013). Furthermore, characterization of phytyl ester synthases previously confirmed that PES is the main contributor to thylakoid decomposition and PG enlargement during chloroplast senescence and nitrogen deprivation through their role in the biosynthesis of fatty acid phytate and triacylglycerol (Lippold et al., 2012). Moreover, a homolog of PES in tomato was found to be involved in the formation of abundant carotenoid esters in chromoplasts, also affecting petal color (Ariizumi et al., 2014). The reduction in accumulation of esterized carotenoids in the *pyp* mutant was therefore expected. Taken together, these findings suggest that *BrPYP* forms more xanthophyll esters during the breakdown of carotenoids.

## Conclusion

In conclusion, the results of this study unequivocally demonstrate that normal yellow petals of *B. rapa* contain more well-developed chromoplasts and PGs, with more esterified carotenoids compared to the pale-yellow mutant. Furthermore, the fine mapping and VIGS findings further suggest that *BrPYP* is the key factor causing higher accumulation of esterified carotenoids in the yellow flowers. Meanwhile, mutation in the promoter of *BrPYP* resulted in down-regulated expression, preventing carotenoid esterification and decreasing the efficiency of carotenoid sequestration within the chromoplast. Overall, these findings highlight the molecular mechanism underlying *BrPYP*-mediated regulation of petal carotenoid pigmentation, and suggest a role of phytyl ester synthase in carotenoid esterification in *B. rapa*.

## Data Availability Statement

Sequencing data has been deposited in the NCBI Sequence Read Archive (SRA) under accession number PRJNA776414.

## Author Contributions

PL, SY, and FZ designed the research. PL carried out sequencing and data analyses, and wrote the manuscript. SL performed the GUS and VIGS experiments. DZ provided the sequencing material. TS, XZ, WW, XX, and YZ provided comments relating to the manuscript. All authors read and approved the final version of the manuscript.

## Conflict of Interest

The authors declare that the research was conducted in the absence of any commercial or financial relationships that could be construed as a potential conflict of interest.

## Publisher’s Note

All claims expressed in this article are solely those of the authors and do not necessarily represent those of their affiliated organizations, or those of the publisher, the editors and the reviewers. Any product that may be evaluated in this article, or claim that may be made by its manufacturer, is not guaranteed or endorsed by the publisher.

## Footnotes

1
https://www.genome.jp/tools-bin/clustalw

2
https://espript.ibcp.fr/ESPript/cgi-bin/ESPript.cgi

3
http://bioinformatics.Psb.ugent.be/webtools/plantcare/html/

4
http://www.metware.cn/

## Supplementary Material

Supplementary Figure 1 The protein sequence and predicted structure of BrBYP. **(a)** Sequence alignment of BrBYP protein between the pale-yellow mutant and yellow type. **(b)** The predicted protein structure of BrBYP.

Supplementary Figure 2 The expression patterns of *BrPDS1* **(a)** and *BrPDS2* **(b)** determined by real-time qRT-PCR.
