# Supplementary figures and images for: The Carotenoid Esterification Gene BrPYP Controls Pale-Yellow Petal Color in Flowering Chinese Cabbage (Brassica rapa L. subsp. parachinensis)
Source: Front Plant Sci. 2022 May 3;13:844140. doi: 10.3389/fpls.2022.844140 (PMC9111173; doi:10.3389/fpls.2022.844140)

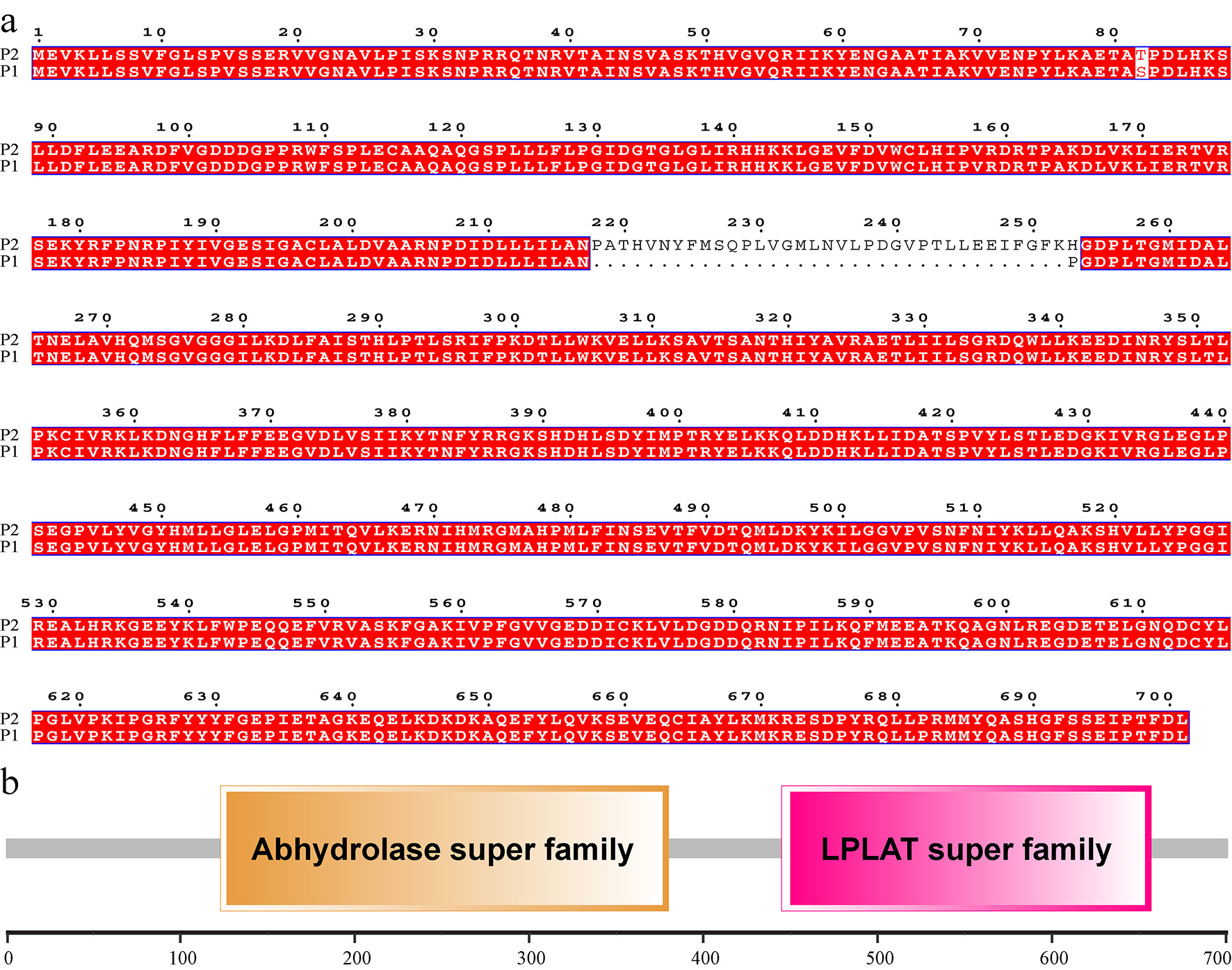

Supplement: Supplementary Figure 1 — The protein sequence and predicted structure of BrBYP. (a) Sequence alignment of BrBYP protein between the pale-yellow mutant and yellow type. (b) The predicted protein structure of BrBYP. [file Image_1.JPEG]

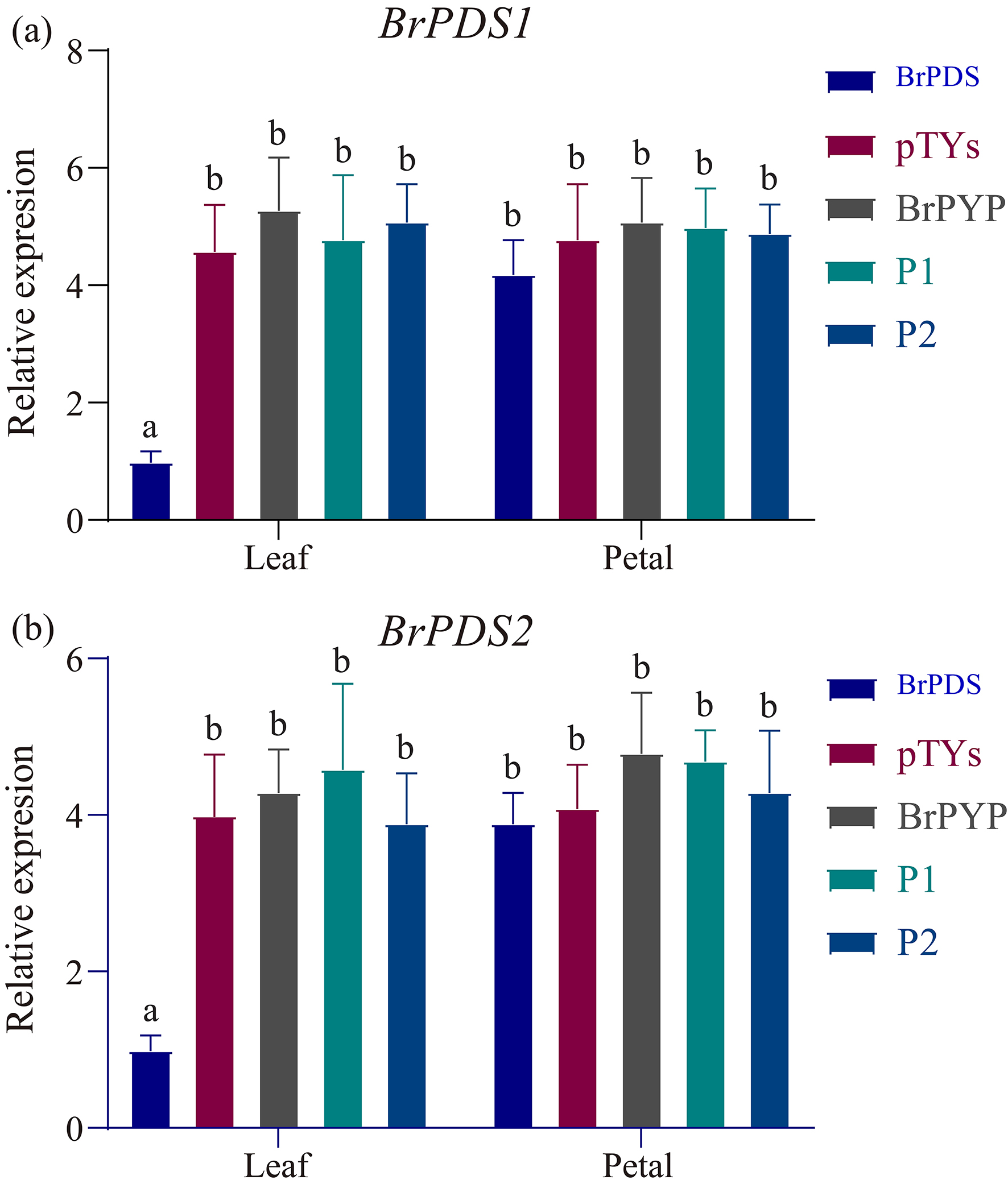

Supplement: Supplementary Figure 2 — The expression patterns of BrPDS1 (a) and BrPDS2 (b) determined by real-time qRT-PCR. [file Image_2.JPEG]
