# Supplementary material for: The Carotenoid Esterification Gene BrPYP Controls Pale-Yellow Petal Color in Flowering Chinese Cabbage (Brassica rapa L. subsp. parachinensis)
Source: Front Plant Sci. 2022 May 3;13:844140. doi: 10.3389/fpls.2022.844140 (PMC9111173; doi:10.3389/fpls.2022.844140)
Supplement: Supplementary file 3 [file Data_Sheet_1.docx]

**Supplementary Table 1. Sequences of the SNP markers.**

| **SNP marker** | **Primer AlleleX** | **Primer AlleleY** | **Primer Common** |
| --- | --- | --- | --- |
| A02_25013868 | GAAGGTGACCAAGTTCATGCTCATTTTAGCTCCTGGGACACTCG | GAAGGTCGGAGTCAACGGATTACATTTTAGCTCCTGGGACACTCA | GCCGGTGGCCAAGCTGAGAGTA |
| A02_25077619 | GAAGGTGACCAAGTTCATGCTCGACGACTGCAGCAGCGG | GAAGGTCGGAGTCAACGGATTGCGACGACTGCAGCAGCGA | CTTGTCGCCGGAGCTATGGCTT |
| A02_25420125 | GAAGGTGACCAAGTTCATGCTAACAAAAAGCACTGGTACAATCTTCAAATA | GAAGGTCGGAGTCAACGGATTCAAAAAGCACTGGTACAATCTTCAAATC | TTAGAATGAGAATCCAAAGAAAGCATGTCAA |
| A02_25538550 | GAAGGTGACCAAGTTCATGCTAAAAATAGTTCCAATTCACTTCTAGTTTTAAG | GAAGGTCGGAGTCAACGGATTGAAAAATAGTTCCAATTCACTTCTAGTTTTAAT | CTTGAACTTTCATAGGATTTTTTATTTCGTTTTTTT |
| A02_25615364 | GAAGGTGACCAAGTTCATGCTTCTGTTCAATCGACGAAAGGAAGAG | GAAGGTCGGAGTCAACGGATTATCTGTTCAATCGACGAAAGGAAGAA | CACATTAGGGTTTTGATTCATGTATTCATCTA |
| A02_25913391 | GAAGGTGACCAAGTTCATGCTCAATATCTTTAGCAGTACCACCAAAAG | GAAGGTCGGAGTCAACGGATTCCAATATCTTTAGCAGTACCACCAAAAA | GATTTATATTTGACTTTTTATTAAACTTCGTTGCAT |
| A02_25939400 | GAAGGTGACCAAGTTCATGCTAGAACCGGATCTTTTGGCTATCTTG | GAAGGTCGGAGTCAACGGATTAGAACCGGATCTTTTGGCTATCTTC | CAAAAGTGCTTCTCCAACCAAACCCAA |
| A02_25962798 | GAAGGTGACCAAGTTCATGCTTGGGCATATCTTCTGCTTCCCG | GAAGGTCGGAGTCAACGGATTGTGGGCATATCTTCTGCTTCCCT | CTCACCGATCAAAAGGTACTGCAGAA |
| A02_26047550 | GAAGGTGACCAAGTTCATGCTTTTATCAAACATCTGTGTGTCCGCC | GAAGGTCGGAGTCAACGGATTTATTTATCAAACATCTGTGTGTCCGCT | TGCTTTTTCTAAACGGCAAAGATGCGTT |
| A02_26064584 | GAAGGTGACCAAGTTCATGCTAACCAACGAGGCGGACCATCAT | GAAGGTCGGAGTCAACGGATTAACCAACGAGGCGGACCATCAA | GACTTCTTGGAGGAAGCGAGAGATTT |
| A02_26057775 | GAAGGTGACCAAGTTCATGCTCTTGCCTGCCACGAGAATAAAATCA | GAAGGTCGGAGTCAACGGATTTGCCTGCCACGAGAATAAAATCG | CTCTTAAACCAGATCAGTCTGTCTTAAAG |
| A02_26096440 | GAAGGTGACCAAGTTCATGCTAACAAACAAATAATAATTTCTGAAACTGGAAG | GAAGGTCGGAGTCAACGGATTAACAAACAAATAATAATTTCTGAAACTGGAAC | CCTTTCAGAGCAAACTCTCGGTTCTTT |
| A02_26340608 | GAAGGTGACCAAGTTCATGCTGAAGAGGCCAAGGGCTAAAGTC | GAAGGTCGGAGTCAACGGATTGAAGAGGCCAAGGGCTAAAGTG | TCATAGTTGTATGTTTTACTGATTCTTATTCAAAA |
| A02_26865674 | GAAGGTGACCAAGTTCATGCTTATACAAGGTGACGTGCAATGCAAG | GAAGGTCGGAGTCAACGGATTCTATACAAGGTGACGTGCAATGCAAT | GAACGCTACATCGTATAGCGACCTTA |
| A02_27292378 | GAAGGTGACCAAGTTCATGCTGTTTGCTCCCTCTTTTATGCCTTTC | GAAGGTCGGAGTCAACGGATTAGTTTGCTCCCTCTTTTATGCCTTTT | TAATCATTGACTTCAAAATTGCAAGAAAAATCAAA |
| A02_27392675 | GAAGGTGACCAAGTTCATGCTTAGTACCAGTATTCAATCTCCTAAAGC | GAAGGTCGGAGTCAACGGATTTAGTACCAGTATTCAATCTCCTAAAGG | GCAGAGACTCAGGGGAGTAGCTAT |

**Supplementary Table 2. Gene-specific and promoter primers of *BraA02g037160.3C* and *BraA02g037170.3C*.**

| Gene | Forward Primer | Reverse Primer | Type |
| --- | --- | --- | --- |
| *BraA02g037160.3C* | CCTGAGTAGCGATTAATAACC | CGGAGCCAAGAAGTGTAG | promoter |
| *BraA02g037160.3C* | ATGGAGGCTACACTTCTTGGCT | TCAGAGATCAAACGTCGGAAT | cds |
| *BraA02g037170.3C* | AGCCTTGGTCTAAACTTCTA | CGATGCTACGGAGTTAATGG | promoter |
| *BraA02g037170.3C* | ATGGAGGTTAAACTACTTAGCTC | TCAGAGATCAAACGTCGGAAT | cds |

**Supplementary Table 3. QPCR primers used for expression analysis.**

| **Gene** | **Forward Primer** | **Reverse Primer** |
| --- | --- | --- |
| *BraA02g037160.3C* | TTTGAGGACGGTGTAGATC | CTGGTAGGCATAATGTAATCTG |
| *BraA02g037170.3C* | GAAAGGGTGAAGAATACAAG | GTCGTCTTCTCCAACAAC |
| *BrPDS1* | CTAATGCCGTTTCAGGACATAGTG | CTCCAGTATCGAACTCACTCTCG |
| *BrPDS2* | AAGGTCCGTGTACAAGACCATCC | ACAGACCGAACTGCACAATAGACT |

**Supplementary Table 4. Statistics of the sequencing data.**

| Code ID | Clean reads | Clean base | Q30 (%) | GC (%) |
| --- | --- | --- | --- | --- |
| P_1_ | 202,531,626 | 30,379,743,900 | 85.81 | 38.77 |
| P_2_ | 208,423,334 | 31,263,500,100 | 88.34 | 41.77 |
| Pale-yellow-bulk | 209,006,868 | 31,351,030,200 | 89.05 | 40.18 |
| Yellow-bulk | 207,592,868 | 31,138,930,200 | 87.83 | 40.2 |

**Supplementary Table 5. Statistics of the mapping to the *B. rapa* reference genome.**

| Code ID | Total reads | Mapped (%) | Properly mapped (%) |
| --- | --- | --- | --- |
| P_1_ | 202,531,626 | 98.12 | 93.19 |
| P_2_ | 208,423,334 | 98.35 | 92.98 |
| Pale-yellow-bulk | 209,006,868 | 98.52 | 94.01 |
| Yellow-bulk | 207,592,868 | 98.57 | 93.69 |

**Supplementary Table 6. Cis-acting regulatory elements in the deleted promoter sequences of *BraA02g037170.3C*.**

| Motifs | Sequence | Number | Organism | Function |
| --- | --- | --- | --- | --- |
| 3-AF3 binding site | CACTATCTAAC | 1 | *Pisum sativum* | Part of a conserved DNA module array (CMA3) |
| MRE | AACCTAA | 2 | *Petroselinum crispum* | MYB binding site involved in light responsiveness |
| TGACG-motif | TGACG | 1 | *Hordeum vulgare* | Cis-acting regulatory element involved in the MeJA-responsiveness |
| STRE | AGGGG | 2 | *Arabidopsis thaliana* | NA |
| chs-CMA1a | TTACTTAA | 1 | *Daucus carota* | Part of a light responsive element |
| ABRE | ACGTG | 2 | *Arabidopsis thaliana* | Cis-acting element involved in the abscisic acid responsiveness |
| TATA | TATAAAAT | 1 | *Arabidopsis thaliana* | NA |
| CAAT-box | CAAAT | 15 | *Pisum sativum* | Common cis-acting element in promoter and enhancer regions |
| TCT-motif | TCTTAC | 1 | *Arabidopsis thaliana* | Part of a light responsive element |
| G-Box | CACGTT | 1 | *Pisum sativum* | Cis-acting regulatory element involved in light responsiveness |
| as-1 | TGACG | 1 | *Arabidopsis thaliana* | NA |
| GT1-motif | GGTTAA | 3 | *Arabidopsis thaliana* | Light responsive element |
| G-box | TACGTG | 2 | *Arabidopsis thaliana* | Cis-acting regulatory element involved in light responsiveness |
| ERE | ATTTCATA | 3 | *Nicotiana glutinos* | NA |
| ABRE4 | CACGTA | 1 | *Zea mays* | NA |
| ARE | AAACCA | 3 | *Zea mays* | Cis-acting regulatory element essential for the anaerobic induction |
| AT~TATA-box | TATATA | 5 | *Arabidopsis thaliana* | NA |
| WRE3 | CCACCT | 1 | *Pisum sativum* | NA |
| MYC | CATTTG | 1 | *Arabidopsis thaliana* | NA |
| MYB-like sequence | TAACCA | 1 | *Arabidopsis thaliana* | NA |
| ACE | CTAACGTATT | 1 | *Petroselinum crispum* | Cis-acting element involved in light responsiveness |
| ABRE3a | TACGTG | 1 | *Zea mays* | NA |
| MYB | TAACCA | 1 | *Arabidopsis thaliana* | NA |
| TATA-box | TATAAA/TATA | 39 | *Helianthus annuus* | Core promoter element around -30 of transcription start |
| CGTCA-motif | CGTCA | 1 | *Hordeum vulgare* | Cis-acting regulatory element involved in the MeJA-responsiveness |
